# Retrospect of Medical Progress

**Published:** 1884-01-20

**Authors:** 


### *RETROSPECT OF MEDICAL PROGRESS.*

It may truthfully be said that there has been decided activity and progress in medical study during the year just past. And if we go back so as to cover a decade of time, the advances in medical science are as unmistakable as they are important.

True it is that a vast amouut of trash and useless material has been published by men whose writings are as empirical as their practice, and thousands of successes are reported which doubtless are due to accident or chance results. Yet, as in the ocean's diurnal flows and ebbs, a few precious shells are always left upon the beach; so in the mass of our medical literature, a few important facts and discoveries remain to enrich our store of knowledge and strengthen the armamentarium of the profession.

The investigations of Koch in relation to the bacillus of tubercle which gave promise of a great and hopeful discovery are yet under advisement, and investigators—though not yet fully assured as to the claims of Koch, are actively engaged in the study of the subject, and the whole field of the germ theory of disease is being explored with promising results.

Touching Listerism in Surgery, it may be said that while the methods of Lister are not now accurately followed, yet the principle is acknowledged as correct, and the cleanliness and improved methods of dressing wounds which have followed, give far better results than formerly prevailed.

In the line of Therapeutics, it may be affirmad that useful and important discoveries have been made, and that America is far in advance of our trans-Atlantic brethren in this department.

In the department of Physiology increased attention is being directed to the Nervous system, in which we appear to be upon the eve of important psycological discoveries. The physiological and real properties of drugs are also undergoing investigation by which many medicinal agents have been removed from an empirical to a scientific position.

In Surgery--especially in abdominal Surgery, greater boldness and improved results have characterized the more recent operations.

In Obstetrics, it is noted that the tendency is to regard puerperal fever as a septic and not a germ disease.

Sanitary science is attracting more and more attention, and the hope prevails that devastating epidemics have had their day, and that, though little has, as yet, been discovered touching the nature of epidemic maladies, we have learned that much may be accomplished in the way of prevention.

